# Profile of Subclinical Psychopathy in Spanish University Students

**DOI:** 10.3390/ijerph19137607

**Published:** 2022-06-22

**Authors:** Carlos Barbosa-Torres, Mónica Guerrero-Molina, Juan Manuel Moreno-Manso, María Elena García-Baamonde, Natalia Bueso-Izquierdo

**Affiliations:** Department of Psychology, University of Extremadura, 06006 Badajoz, Spain; carlosbarbosa@unex.es (C.B.-T.); jmmanso@unex.es (J.M.M.-M.); mgarsan@unex.es (M.E.G.-B.); nbueso@unex.es (N.B.-I.)

**Keywords:** subclinical psychopathy, gender, age, number of intimate relationships, university students

## Abstract

Psychopaths are portrayed as deceitful, manipulative, domineering and narcissistic; the result of an irregular and irresponsible interpersonal style that harms both the psychopath him/herself and others. Furthermore, psychopathy is frequently associated with both violent and antisocial conduct. However, subclinical psychopaths are known for manifesting this type of profile without committing crimes. The objective of this study is to examine the differences that exist in subclinical psychopathy concerning gender, the number of intimate relationships and the age of the university student. The number of university students participating was 1289. They were given the Integrated/Subclinical Psychopathy Questionnaire (CUPIS). The results show that, in subclinical psychopathy, men present higher scores than women and younger persons have higher scores than older persons. In addition, the scores in psychopathy are higher for students who have had a larger number of intimate relationships. The contributions of this study allow us to determine the profile associated with subclinical psychopathy.

## 1. Introduction

Classic authors [1] attribute the harm psychopaths cause to themselves and others to their shallow, capricious and daring character traits, instead of through aggressive, cruel or malicious characteristics. Psychopathy can be defined in terms of poorly developed affection, dominant and narcissistic character traits, in which an irregular and irresponsible interpersonal style of behaviour towards both themselves and others predominates, as well as an antisocial lifestyle [2]. For other authors [3], psychopaths are apparently psychologically healthy persons who are characterised as being friendly and attentive and who behave in a socially open way. However, we can find such traits as impulsiveness, accompanied by licentious acts in their daily dealings. Other research studies state that they are unable to feel empathy or to form normal relations with other persons irrespective of how important they may be, and this can be particularly harmful in the context of sentimental or romantic relationships [4]. Thus, focusing on their emotional reactions and relationships, they maintain superficial relationships, showing a deficit in the recognition of negative emotions in comparison to the general population [5].

Psychopathy is associated with both violent and antisocial conduct, although not all those suffering from psychopathy present this type of behaviour [1]. Furthermore, psychopaths are frequently described as natural liars with a great capacity for manipulation [6,7]. Authors [8] point out that criminal psychopaths can be differentiated from subclinical psychopaths. The main difference between both groups lies in the fact that the former commit crimes of some type and degree [9,10]. Thus, subclinical psychopaths, despite the abovementioned characteristics, do not commit crimes. In this sense, one study [9] concluded that criminal psychopaths show psychopathic characteristics to a greater degree than subclinical psychopaths do, especially so far as affective and antisocial characteristics are concerned. It can, therefore, be said that criminal and non-criminal psychopaths mainly differ in the degree of expression of psychopathic traits [11].

Part of the literature describes the existence of differences with respect to gender, in which males show higher scores in psychopathic traits than women [12]. In the same way, these traits have been studied from childhood to adulthood [13], where it is shown that psychopathic traits decrease with age, although these persons can continue to present high rates of psychopathic traits as adults. Despite this fact, other research studies show that psychopathy can manifest itself in different age ranges and in both sexes [14]. However, other authors [15,16] postulated that these variables could function as sampling artefacts that affect the analysis of the results; so, it is recommended to avoid putting both genders together in studies on psychopathy, specifying that the results of a joint analysis would confuse the personality and group differences, which would lead to reappraising the results of many works of research.

The research carried out on non-clinical populations has shown that the number of intimate relationships is related to the duration of the sentimental relationship and thus to the different styles of relationship or sexual partnerships [17]. This fact is magnified in subjects with high scores in psychopathy since they show a great interest in having diverse affective-sex partners, as well as in sporadic sex or even promiscuous sexual activities [18]. Similarly, it has also been associated with the use of coercive physical and/or verbal methods in intimate relationships, and this affects the quality of the relationship, decreasing the amount of commitment [19].

Due to the few existing and updated investigations on this subject, the main implication of the study consists of the identification of a profile of people with psychopathic personality traits who are integrated into society and who, therefore, present different characteristics and ways of relating to their affective-social environment. Owing to the data presented, the objective of this study is to examine the existing differences in psychopathy with respect to gender, the number of intimate relationships and the age of the students of the University of Extremadura. We thus pose the ideas that there are significant differences in psychopathy with respect to the gender of the students (Hypothesis 1); the number of intimate relationships (Hypothesis 2); that age is also related to subclinical psychopathy (Hypothesis 3).

## 2. Method and Materials

### 2.1. Participants

The sample under study consisted of 1289 university students at the University of Extremadura (UEx). The research was carried out during the academic year 2020/2021. The sample was made up of university students in the first to the fourth years, studying existing degrees in different areas of knowledge on the campuses of Badajoz and Caceres, with 37.8% of participants from the Faculty of Education (*n* = 487), 26.7% from the Faculty of Medicine (*n* = 344) and 35.5% from the Faculty of Law (*n* = 458). The participants in the study came from families with an average socioeconomic level as far as studies, income and work situation are concerned. In total, 56.6% were females between 17 and 57 years of age (*M* = 22.38; *DT* = 4.81); 51.3% (*n* = 662) currently had a partner and 45.6% did not (*n* = 588); 2.9% were married (*n* = 37) and 0.2% separated (*n* = 2). As for the number of previous partners, 58.3% (*n* = 751) had previously had 1 or 2 intimate relationships. This was followed by the percentage of students who had had no previous relationships (17.4%), those who had had 3 or 4 prior relationships (13.7%), and those who had had more than 4 previous intimate relationships (10.6%).

### 2.2. Instruments

*Integrated or Subclinical Psychopathy Questionnaire* (CUPIS) [10]. This is an instrument to evaluate the subclinical psychopathy in the general population over 17 years of age. The CUPIS is based on the detailed study of the English language literature on the subject and was elaborated on the basis of already existing questionnaires in English as well as a review of the classical authors in the literature [1,8], upon which almost the entirety of the research studies and questionnaires in existence are based. It consists of 60 items structured into 4 main factors, each made up of 15 items that, in turn, are divided into 12 facets or subscales, each one made up of 5 items. A Likert-type scale of 5 points is used to evaluate the degree of agreement and disagreement with respect to each one of the items («I can change at will the impression I give of myself depending on who I am speaking to» and «I tell convincing and credible stories, although it is fairly improbable that they could actually happen»). The internal consistency found in the data from our study is 0.76.

### 2.3. Procedure

First of all, we made a formal agreement with the Academic Secretaries of the selected Faculties of the UEx, informing them of the content of the project. Following the issue of the pertinent licences necessary for implementing the tests, the evaluation instruments were applied during the academic period 2020/2021.

The tests were administered collectively in a single session in which the participants were given the questionnaires along with the instructions. The objective of the research was explained, ensuring both anonymity and confidentiality of the results obtained from the tests. The evaluators were present at all times during the implementation of the tests to resolve any doubts and to ensure that the questionnaires were adequately completed. The time taken to complete the tests was between 20 and 30 min and no great problems with understanding occurred.

The information was collected and the students’ responses were revised in each of the protocols based on atypical contents in the responses. No atypical contents were found and no questions were found to be unanswered, with the exception of two questionnaires that were eliminated.

All procedures performed were in accordance with the ethical standards of Extremadura University (Ref.: 181/2020) and with the 1964 Helsinki declaration and its later amendments or comparable ethical standards. All subjects gave their informed consent for inclusion before they participated in the study.

### 2.4. Data Analysis

The Statistical Package for the Social Sciences, SPSS, version 25, was used for the statistical treatment of the data. The considered confidence interval was set at 95%.

Having determined that the use of parametric tests was appropriate with respect to the nature of the variables and the sample size (*n* = 1289), we carried out an inferential analysis to determine whether there were statistically significant differences in subclinical psychopathy with respect to gender and the number of intimate relationships of the university students. To be precise, we used the Welch *t*-test to compare the averages in two independent samples with unequal variances. 

We also carried out a Pearson’s correlation to analyse the relationship between subclinical psychopathy and the students’ ages. We then carried out a linear regression analysis to determine the extent to which gender, the number of partners and the age of the university students can predict the facets of subclinical psychopathy.

## 3. Results

In order to discover whether the subclinical psychopathy of university students is influenced by gender, we carried out an analysis of the averages (Table 1).

There are significant differences in both the global scale of subclinical psychopathy and those that make up the construct with respect to the gender of the students. In such a way, male university students show a greater number of psychopathic traits than female students in all the analysed variables (*p* < 0.001).

Below we show the results obtained concerning subclinical psychopathy and the number of intimate relationships in university students through the Welch *t*-test (Table 2).

As we can see from Table 2, there are significant differences in all the factors and facets of subclinical psychopathy with respect to the number of intimate relationships (*p* < 0.001). In this sense, it must be stressed that the scores in psychopathy are higher in students with a higher number of intimate relationships.

As for the correlation analysis between subclinical psychopathy and age, the results indicate the following (Table 3).

The results show evidence of a negative correlation between age and the global scale of subclinical psychopathy (*r* = −0.158; *p* < 0.001), as well as in all the factors and subscales analysed, except for the scale referring to *Superficial affection* and *indifference*, in which there is no evidence of a significant relationship with respect to the age of the young university students (*r* = −0.045; *p* = 0.109). Therefore, among these university students, the evidence would seem to indicate that the higher the age, the lower the score in subclinical psychopathy.

To determine how far gender, the number of affective relationships and age can significantly predict the facets of subclinical psychopathy, we carried out a regression analysis (Table 4).

Based on the results obtained, gender (0 = *male*; 1 = *female*) allows us to significantly predict subclinical psychopathy (β = −0.421; *p* < 0.001); so, being a man is significantly related to the facets of subclinical psychopathy. In this sense, it is important to point out that gender explains 17.7% of the variance in the global scale of subclinical psychopathy.

Similarly, the number of affective partners of the university students allows us to predict subclinical psychopathy (β = 0.236; *p* < 0.001), explaining 5.6% of the variance in the global scale of subclinical psychopathy. Thus, it can be stated that a greater number of affective relationships are related to facets of subclinical psychopathy.

Finally, the results show that age explains 2.5% of the variance of the responses in the global scale of subclinical psychopathy (β = −0.158; *p* < 0.001); so, age allows us to predict facets of subclinical psychopathy, although it should be said that age does not contribute to predicting *Superficial affection* and *indifference* (β = −0.045; *p* = 0.109).

## 4. Discussion

The objective of the present study was to examine the existing differences in psychopathy with respect to gender, the number of intimate relationships and the age of the university students. In this sense, it can be confirmed that these variables tend to significantly predict facets of subclinical psychopathy among young university students.

As for gender, the conclusion is that there are significant differences as the male students present a higher number of psychopathic traits than the female students. Previous research works have obtained these same results in samples of both university and general populations [20,21]. However, some works of research found no significant differences in these traits with respect to gender [22], for instance, a study proposed by Mayer [23] concerning subclinical psychopathy, which predicted affective anxiety with psychopathic traits linked to interpersonal and affective difficulties for both genders equally. The authors defined these behaviour patterns as a fear of feeling emotions, or a fear of intimacy and emotional closeness, making them want to get away from people.

Concerning the above, the fact that certain psychopathic traits are more prevalent than others depends more on the structure of the personality, the emotions and the lifestyle and less on gender [10]. Furthermore, it is possible that the research works that include the gender variable may be influenced by the stereotypes linked to gender [24], as the great majority of studies usually approach this question through arguments based on the standards of behaviour in intimate relationships linked to the influence of the patriarchal model [25]. That is why the majority of studies that present differences based on gender limit themselves to specifying the statistical significance without explaining what the said differences consist of or their possible origin [26].

As for the question of age, significant negative correlations are obtained for all the factors and subscales, by which the younger students show more psychopathic traits than the older students. These data are close to those of a study [27] involving adolescents and young people, in which the youngest interval, from 17 to 18 years of age, is the one that has the highest scores in psychopathy. Another study with a range from 18 to 34 years of age did not show significant differences with respect to gender but did show such differences with respect to age. In this sense, they found a significant negative relation between age and all the facets of psychopathy, i.e., the greater the age, the lower the score in psychopathy [28]. Our data are also close to those of another study carried out on the general population, in which the maximum scores were obtained by the age group from 20 to 24 years of age [29]. 

One of the possible explanations could be generational changes, as the circumstances that condition the growth and development of older persons are very different from those of younger persons, a fact that is reflected in all spheres of life [30]. 

As for the number of relationships, significant differences were obtained between this variable and subclinical psychopathy. This evidence suggests that the greater the number of intimate relationships, the more psychopathic traits the students present. These results are in line with other studies [31], which underline the fact that a psychopath will have a greater number of intimate relationships and an open attitude towards casual sex. This may be due to their capacity for exploiting and manipulating others, as well as their egocentric characteristics. In this sense, previous works of research have postulated that the sexual orientation or promiscuous activity is a predictor of psychopathy [8]. 

However, classical authors [1] have traditionally defended the idea that a greater number of intimate relationships characterised by an interpersonal or frivolous sexual life, as well as frequent relations with a short duration, are not sufficient conditions for classifying anyone as a psychopath, even though they do form part of the defining characteristics of psychopathy.

One study [32], carried out with 884 participants between 18 and 74 years of age, reached the conclusion that psychopathy is a strong predictor of infidelity in romantic relationships. The principal repercussion of this type of behaviour is that it generates personal distress and insecurity concerning the bonding in the partner [33]. Consequently, it produces a high level of stress and dissatisfaction, as well as a higher rate of violence within the couple [34]. The characteristics that define psychopaths, together with the distress present in the partner, could be precursors of early sexual activity, infidelities and, as part of this, the existence of a greater number of intimate relationships [35]. It is precisely because of all the above that there are authors who consider that longer relationships over time could lessen some of the symptoms of psychopathy [36].

Despite the conclusions described, the present research is not without its limitations. In this sense, it should be pointed out that the sample is not equivalent in the different age ranges, as there is a predominance of young subjects due to the fact that the sample is of students from the University of Extremadura, a fact which impedes the possible generalisation of the results to other age ranges. Furthermore, a transversal methodology has been used, which makes it impossible to have a temporal evolution of the scores and establish causal relations between the analysed variables. The self-reporting measures for the data are tests that lose solidity as far as objectivity is concerned since participants frequently respond in a socially acceptable manner. For future research, it would be convenient to be able to relate to the profile of subclinical psychopathy possible associated events, such as abuse or neglect, childhood trauma, recent and stressful life events, consumption of illicit substances, or other prevailing personality traits.

## 5. Conclusions

In conclusion, our study provides an important contribution since it allows us to determine the psychopathy profile of people who are integrated into society in a sample of Spanish students. The results show that men present more psychopathic traits than women [20,21], specifically in interpersonal, interactive, affective–emotional styles and erratic lifestyles. In relation to age, older people have lower scores in subclinical psychopathy, although it seems to be unrelated to superficial affection and indifference [27,28]. In addition, psychopathy scores are higher in students who have had a greater number of intimate relationships, which is related to erratic lifestyles that make their relationships suffer [8,32].

## Figures and Tables

**Table 1:** Comparison of averages in subclinical psychopathy with respect to gender.

	Male		Female		*t*
	*M*	*SD*	*M*	*SD*	
Factor I: Interpersonal style	2.66	1.071	1.80	0.857	15.567 ***
Superficial charm and loquacity	2.72	1.027	1.89	0.854	15.389 ***
False appearances	2.44	1.165	1.71	0.890	12.409 ***
Egocentric self-image	2.59	1.157	1.84	0.929	12.553 ***
Factor II: Interactive style	2.13	1.110	1.54	0.748	10.815 ***
Repetitive lies and falsehood	2.30	1.151	1.63	0.807	11.679 ***
Deception and lack of scruples	2.14	1.113	1.61	0.785	9.578 ***
Manipulation, control and commodification	1.89	1.074	1.39	0.713	9.398 ***
Factor III: Affective-emotional style	2.39	1.020	1.54	0.746	16.532 ***
Superficial affection and indifference	2.59	1.049	1.68	0.831	16.840 ***
Insensitivity and cruelty	2.25	1.083	1.48	0.776	14.256 ***
Lack of remorse/guilt	2.10	1.073	1.51	0.763	11.152 ***
Factor IV: Erratic lifestyle	2.37	1.105	1.51	0.790	15.492 ***
Parasitism and opportunism	2.20	1.048	1.50	0.779	13.179 ***
Unstable interpersonal relationships	2.09	1.112	1.46	0.760	11.586 ***
Impersonal and frivolous sex life	2.63	1.239	1.61	0.922	16.261 ***
Global scale	2.54	1.076	1.66	0.817	16.085 ***

*Note*: *** *p* < 0.001.

**Table 2:** Comparison of the averages in subclinical psychopathy with respect to the number of partners.

	0–2 Partner Relationships		3 or More Partner Relationships		*t*
	*M*	*SD*	*M*	*SD*	
Factor I: Interpersonal style	2.04	0.953	2.62	1.194	−7.868 ***
Superficial charm and loquacity	2.12	0.921	2.64	1.194	−7.060 ***
False appearances	1.89	0.993	2.44	1.232	−7.129 ***
Egocentric self-image	2.04	1.033	2.53	1.213	−6.333 ***
Factor II: Interactive style	1.70	0.883	2.10	1.143	−5.564 ***
Repetitive lies and falsehood	1.82	0.940	2.24	1.201	−5.572 ***
Deception and lack of scruples	1.76	0.922	2.09	1.098	−4.735 ***
Manipulation, control and commodification	1.49	0.808	1.96	1.137	−6.726 ***
Factor III: Affective-emotional style	1.80	0.866	2.25	1.180	−6.128 ***
Superficial affection and indifference	1.98	0.943	2.38	1.233	−5.294 ***
Insensitivity and cruelty	1.69	0.906	2.18	1.167	−6.755 ***
Lack of remorse/guilt	1.70	0.850	1.98	1.208	−3.918 ***
Factor IV: Erratic lifestyle	1.74	0.899	2.33	1.261	−7.683 ***
Parasitism and opportunism	1.71	0.891	2.10	1.132	−5.593 ***
Unstable interpersonal relationships	1.61	0.845	2.12	1.240	−6.820 ***
Impersonal and frivolous sex life	1.90	1.070	2.54	1.375	−7.552 ***
Global scale	1.91	0.936	2.44	1.209	−7.109 ***

*Note*: *** *p* < 0.001.

**Table 3:** Correlation between subclinical psychopathy and age.

	Age
Factor I: Interpersonal style	−0.095 **
Superficial charm and loquacity	−0.064 *
False appearances	−0.100 ***
Egocentric self-image	−0.127 ***
Factor II: Interactive style	−0.138 ***
Repetitive lies and falsehood	−0.072 *
Deception and lack of scruples	−0.174 ***
Manipulation, control and commodification	−0.119 ***
Factor III: Affective-emotional style	−0.130 ***
Superficial affection and indifference	−0.045
Insensitivity and cruelty	−0.124 ***
Lack of remorse/guilt	−0.156 ***
Factor IV: Erratic lifestyle	−0.165 ***
Parasitism and opportunism	−0.158 ***
Unstable interpersonal relationships	−0.165 ***
Impersonal and frivolous sex life	−0.144 ***
Global scale	−0.158 ***

*Note*: * *p* < 0.05; ** *p* < 0.01; *** *p* < 0.001.

**Table 4:** Regression analysis between subclinical psychopathy with respect to gender, number of partners and age.

	Gender			Number of Partners			Age		
	*R*²	β	*t*	*R*²	β	*t*	*R*²	β	*t*
Factor I: Interpersonal style	0.166	−0.408	−16.020 ***	0.045	0.213	7.804 ***	0.009	−0.095	−3.432 **
Superficial charm and loquacity	0.162	−0.402	−15.763***	0.040	0.199	7.278 ***	0.004	−0.064	−2.297 *
False appearances	0.114	−0.337	−12.844 ***	0.040	0.200	7.317 ***	0.010	−0.100	−3.614 ***
Egocentric self-image	0.115	−0.339	−12.913 ***	0.034	0.185	6.768 ***	0.016	−0.127	−4.605 ***
Factor II: Interactive style	0.091	−0.302	−11.362 ***	0.030	0.174	6.321 ***	0.019	−0.138	−5.015 ***
Repetitive lies and falsehood	0.104	−0.322	−12.214 ***	0.030	0.174	6.344 ***	0.005	−0.072	−2.572 *
Deception and lack of scruples	0.072	−0.269	−10.009 ***	0.026	0.160	5.828 ***	0.030	−0.174	−6.350 ***
Manipulation, control and commodification	0.071	−0.266	−9.890 ***	0.038	0.195	7.134 ***	0.014	−0.119	−4.309 ***
Factor III: Affective-emotional style	0.187	−0.432	−17.202 ***	0.042	0.204	7.478 ***	0.017	−0.130	−4.722 ***
Superficial affection and indifference	0.190	−0.435	−17.354 ***	0.032	0.178	6.475 ***	0.002	−0.045	−1.602
Insensitivity and cruelty	0.147	−0.383	−14.872 ***	0.042	0.205	7.503 ***	0.015	−0.124	−4.483 ***
Lack of remorse/guilt	0.095	−0.309	−11.643 ***	0.023	0.153	5.536 ***	0.024	−0.156	−5.680 ***
Factor IV: Erratic lifestyle	0.169	−0.411	−16.164 ***	0.073	0.270	10.063 ***	0.027	−0.165	−5.995 ***
Parasitism and opportunism	0.127	−0.356	−13.686 ***	0.039	0.196	7.189 ***	0.025	−0.158	−5.725 ***
Unstable interpersonal relationships	0.103	−0.321	−12.151 ***	0.063	0.250	9.263 ***	0.027	−0.165	−6.019 ***
Impersonal and frivolous sex life	0.181	−0.426	−16.886 ***	0.065	0.255	9.467 ***	0.021	−0.144	−5.202 ***
Global scale	0.177	−0.421	−16.662 ***	0.056	0.236	8.714 ***	0.025	−0.158	−5.722 ***

*Note*: * *p* < 0.05; ** *p* < 0.01; *** *p* < 0.001.
